# Development of novel lipidic particles for siRNA delivery that are highly effective after 12 months storage

**DOI:** 10.1371/journal.pone.0211954

**Published:** 2019-02-08

**Authors:** Daniel Clarke, Adi Idris, Nigel A. J. McMillan

**Affiliations:** Menzies Health Institute Queensland, School of Medical Science, Griffith University, Southport, Australia; University of Tennessee Health Science Center, UNITED STATES

## Abstract

Liposomes are versatile and well-proven as a means to deliver nucleic acids into cells. Most of the formulation procedures used are labour intensive and result in unstable end products. We have previously reported on the development of a simple, yet efficient, hydration-of-freeze-dried-matrix (HFDM) method to entrap siRNA within lipid particles. Here we show that the particles are stable up to 12 months after storage at room temperature (RT), 4°C or -20°C. While RT storage results in changes in particle size and polydispersity, gene silencing of all particles was similar to freshly prepared particles following storage for 3, 6, 9 or 12 months at all temperatures. This is the first report of such long-term stability in siRNA-loaded liposomes.

## Introduction

Various methods for formulating polynucleotide-loaded PEGylated particles have been reported to date, including post-insertion [1], reverse-phase evaporation [2], detergent dialysis [3] and ethanol dialysis [4–6]. However, most of these methods, though effective, require relatively complicated and lengthy formulation procedures with the resulting particles suspended in an aqueous state. This has led to long term storage issues including aggregation and/or fusion of the particles [7, 8], hydrolysis of the lipids [7], and instability of siRNA nucleotides in an aqueous environment. Moreover, these formulations are also prone to be affected by stresses occurred during transport, such as agitation or temperature fluctuation [8, 9]. These problems, along with the significantly increased effort required for large-scale production of these particles using the existing formulation procedures has held back clinical development and adoption.

To address these issues, we developed a novel method to formulate stable, siRNA-loaded, PEGylated lipid particles using the hydration of freeze-dried matrix (HFDM) method[10]. This simple, yet-efficient, procedure results in lipid particles that are have highly favourable characteristics for *in vivo* delivery. Indeed, these particles have been used for *in vivo* systemic delivery in animal models to target a range of cancer-related genes, which have resulted in significant tumour elimination and increased survival [11, 12]. HDFM lipoplexes have also been used to reduce gene expression in lung tissues [13], the peritoneal cavity via intraperitoneal delivery [14] and in the vaginal epithelia with the aid of a novel aliginate matrix to increase retention time [15].

The simple mixing of lipids (DOTAP, cholesterol and PEG2000-C_16_Ceramide–molar ratio of 45:45:10) dissolved in *tert*-butanol with nucleic acids dissolved in sucrose followed by freeze-drying and rehydration results in lipid particles that are isotonic and ready for direct *in vivo* injection. Particles are ~190nm in size with a polydispersity index of 0.32 and an overall charge of 45mV. The encapsulated siRNA was protected from serum [10] and show excellent pharmacokinetics with T1/2 z>40h compared to other systems such as galactosylated cationic liposomes (T1/2 z>1h)[16], PEGylated polyplexes (T1/2 z>1.5h)[17], or SNALPS (T1/2 z>6.5h)[18]. This represented an advance in lipid formulation of siRNA for *in vivo* use.

As mentioned above, liposomes are prepared fresh for *in vivo* use as they aggregate, fuse or hydrolyse in aqueous solutions over time. As the HFDM method results in a dried matrix we postulated that these resulting lipoplexes would be highly stable over time. Indeed, our initial studies examined the longevity of the freeze-dried siRNA/lipid matrix stored at 4°C, or room temperature (RT), for 4 weeks and showed that silencing was still significant at this time [10]. Here, we have extended these studies out to 12 months and looked at a range of different storage conditions. We examined the physical characteristics of the particles and their ability to silence target genes *in vitro* at various time points. Our data show that HFDM lipoplexes are highly stable and still active for *in vitro* silencing even 12 months post-production. Such post-production longevity has never previously been reported and represents a significant advance in the field.

## Materials and methods

### Cells and siRNA

HeLa cells were originally obtained from the American Type Culture Collection (ATCC) and were cultured as described previously [19]. The siRNA used in this study was Lamin A/C siRNA [20] obtained from Genesearch (Shanghai, China). siGlo red (Dharmacon, Lafayette, CO) was used as a qualitative transfection indicator as transfection efficiency.

### Liposome formulations

Lipoplexes were prepared by Hydration of Freeze-Dried Matrix (HFDM) method as described previously [10]. Required amounts of DOTAP, cholesterol and PEG2000- C16Ceramide were dissolved in 1 mL of tert-butanol at a molar ratio of 45:45:10. 40μg of siRNA was added to 1 mL of filtered sucrose solution before mixing with the lipid solution. The resultant formulation was then snap-frozen and freeze-dried overnight (ALPHA 1–2 LDplus, Martin Christ, Germany) at a condensing temperature of −80°C and pressure of less than 0.1 mbar. Distilled H2O was then added to the lyophilised product with gentle shaking. A Nitrogen/Phosphate (N/P) ratio of 4:1 was used for all formulations and three separate batches were made for each formulation condition (n = 3). The final product contained 40μg siRNA in 300μL isotonic sucrose solution.

### Particle characterisation

Size, polydispersity and zeta potential of the resultant lipoplexes were measured using a Zetasizer Nano ZS (Malvern Instruments, Malvern, UK) following appropriate dilution in distilled water. Measurements were carried out at room temperature (RT), 4°C and -20°C with 10 runs per measurement undertaken.

### siRNA entrapment efficiency

siRNA entrapment efficiencies were determined using the Quant-iT PicoGreen reagent (Invitrogen, California USA) as previously described [10].

### Lipoplex cell uptake analysis

Cells were forward-transfected with 40μM of siGlo red. Cell uptake efficiency was assessed by flow cytometry 24h post-transfection on a BD LSR FORTESSA cell analyser (BD bioscience, San Jose, CA).

### *In vitro* siRNA transfection

*In vitro* siRNA transfection of liposome-complexed siRNA was performed as previously described [10]. HeLa cells were seeded the day before the transfection experiment at a density of 75,000 cells per well in a 6 well plate. A final concentration of 40 nM liposome-entrapped siRNA suspended in primocin-free complete media was then added to each well. siRNAs were left on cells for 8 h, and cells were then incubated in primocin-containing media for 3 days at 37°C. The level of gene knockdown was determined by qPCR analysis and percentage of knockdown efficiency calculated as previously described [21].

### qPCR

Extract of RNA, cDNA generation, and qPCR were carried out as described previously [19]. Real time primers used: Lamin A/C forward 5’-TGGAGATGATCCCTTGCTGA-3’, Lamin A/C reverse 5’-GCATGGCCACTTCTTCCCA-3’, β-actin forward 5’-AGCCTCGCCTTTGCCGA-3’and β-actin reverse 5’-CTGGTGCCTGGGGCG-3’.

### Statistical analysis

All data were subjected to two-way ANOVAs with Dunnett's post-hoc analyses using GraphPad Prism 7.

## Results and discussion

**Fig 1:**
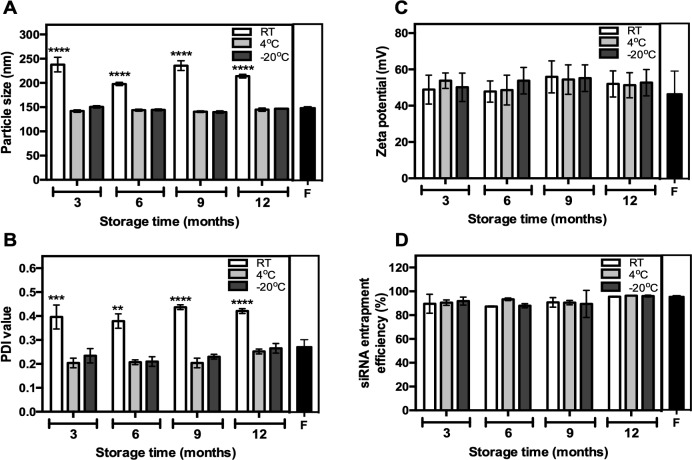
Lipoplex size, polydispersity, zeta potential and siRNA entrapment efficiency remains unchanged with long term storage at various temperatures except the lipoplex size and the polydispersity at room temperature. (A) Size, (B) polydispersity and (C) zeta potential of the resultant lipoplex were measured using a Zetasizer Nano ZS following appropriate dilution in distilled water. Formulations were stored at either -20°C, 4°C or room temperature (RT) and 2 separate batches were made for each condition. (n = 2). (D) siRNA entrapment efficiencies were determined using the Quant-iT PicoGreen reagent. Tests were performed on freshly prepared lipoplex (F–Black shaded bars), and lipoplex stored at various temperatures for different lengths of time post manufacture. Bar graphs represent the mean and the error bars represent the standard deviation. **p value < 0.005, ***p value < 0.0005, ****p value <0.0001, two-way ANOVA using a Dunnett's post-hoc analysis against freshly prepared lipoplexes.

We have previously showed that storage of our formulated lipoplexes for 1 month at RT and 4°C did not affect its physiochemical properties [10]. However, the physiochemical stability of the lipoplexes have never been tested outside these storage conditions. The stability of dried-state lipoplexes was investigated up to 12 months post-manufacture. Following formulation, the particles were stored at either RT, 4°C or -20°C. When compared to freshly-prepared lipoplexes, lipoplex powder stored at RT displayed greater variation in both size and PDI upon rehydration, likely due to varying humidity levels during storage (Fig 1A and 1B). More importantly, no variation in the surface charge (or zeta potential) of particles was observed when stored at any temperature over a 12-month period (Fig 1C), which is important for the stability of the particles and remaining free from agglutination when delivered into the bloodstream. In contrast to what we previously observed [10], the siRNA entrapment efficiency did not fluctuate over time at different temperatures when compared to freshly-prepared lipoplexes, indicating the siRNA encapsulated in the lipoplexes were stable (Fig 1D). Overall, there was minimal effect of the storage time and temperature on the lipoplex physiochemical properties and the stability of the siRNA-loaded lipoplexes, with 4°Cand -20°C showing superior stability in terms of size, charge and size variation.

**Fig 2:**
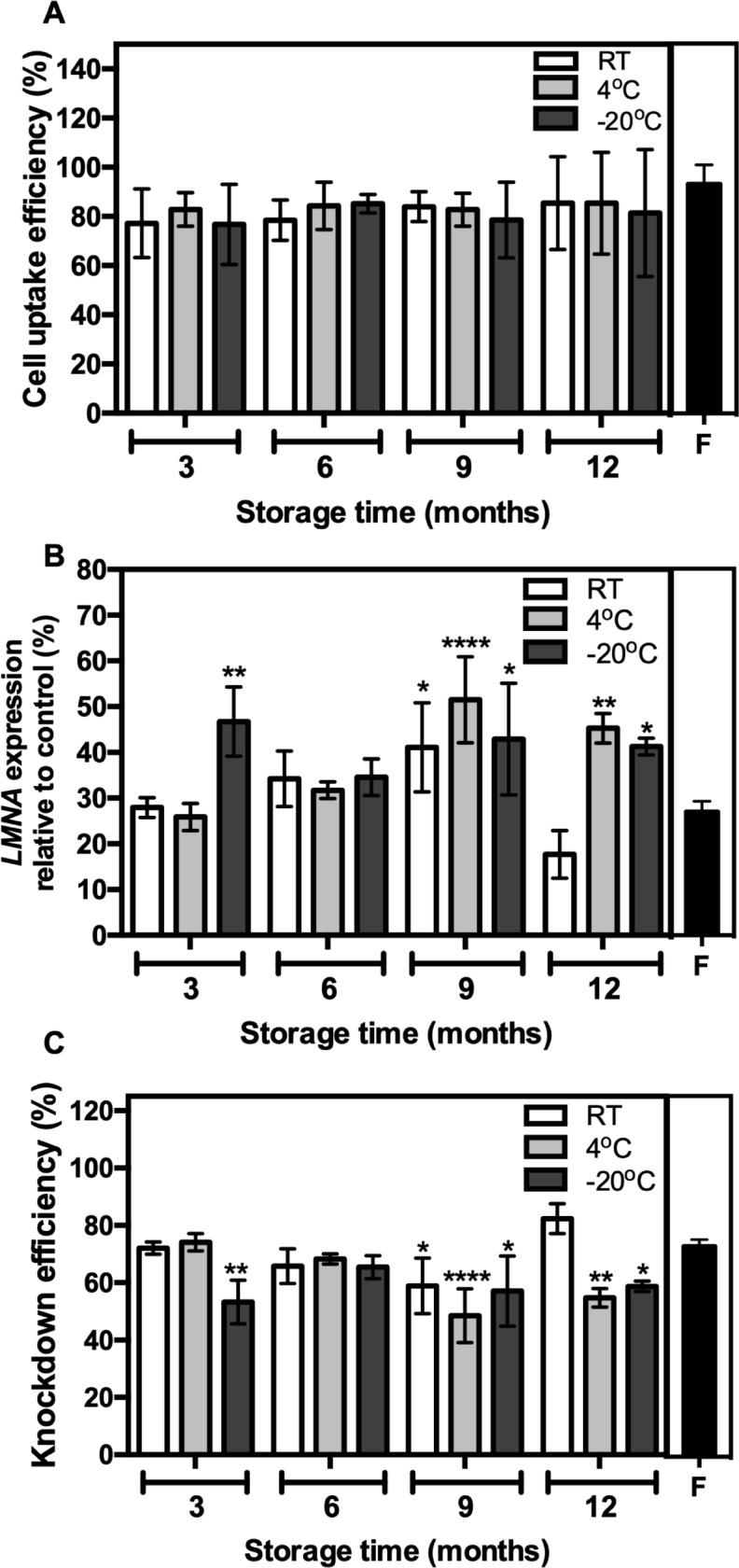
siRNA-mediated target gene knockdown capability was maintained using lipoplexes stored at different temperatures over time *in vitro*. (A) HeLa cells were treated with a final concentration of 40 nM of siGlo complexed into lipoplexes and incubated at 37°C for 24h before assessing siGlo uptake (%) by flow cytometry. Tests were performed on freshly prepared lipoplexes (F–Black shaded bars), and lipoplexes stored at various temperatures for different lengths of time post manufacture. Data is presented as percentage sum of fluorescence intensity. Bar graphs represent the mean and the error bars represent the standard deviation from three independent experiments. (B) HeLa cells were treated with a final concentration of 40 nM of Lamin A/C (*LMNA)* siRNA complexed into lipoplexes and incubated at 37°C for 3 days. Expression levels were calculated relative to non-transfected cells (control) by qPCR analysis and data presented as percentage of *LMNA* expression compared to control. β-actin was used as housekeeping control gene. (C) Percentage of knockdown efficiency was also determined as previously described [21]. Tests were performed on freshly prepared lipoplexes (F–Black shaded bars), and lipoplexes stored at various temperatures for different lengths of time post manufacture. Bar graphs represent the mean and the error bars represent the standard deviation from three independent experiments. **p value < 0.005, ***p value < 0.0005, ****p value <0.0001, two-way ANOVA using a Dunnett's post-hoc analysis against freshly prepared lipoplexes.

To assess whether storage time and temperature have any effect on the functional capability of the lipoplexes, the gene knockdown ability of siRNA-loaded lipoplexes was tested *in vitro*. *In vitro* cell uptake efficiency of lipoplexes stored over time at varying temperatures were comparable to that of freshly prepared lipoplexes (Fig 2A). Liposome powders were rehydrated at each time point, complexed with siRNAs and applied to HeLa cells to determine knockdown of the non-essential structural protein, lamin A/C (*LMNA*). qPCR analysis revealed notable decrease of *LMNA* mRNA expression in HeLa cells treated with *LMNA*-specific siRNA compared to untransfected cells, irrespective of the storage conditions (Fig 2B). Furthermore, the knockdown efficiency (Fig 2C) at the mRNA level was comparable to that of freshly prepared liposomes suggesting that storage time and temperature have minimal effect on the gene-silencing efficiency of the siRNAs-complexed with lipoplexes. Although some liposome preparation groups had significantly different *LMNA* mRNA knockdown levels when compared to freshly prepared liposomes (Fig 2B and 2C), overall knockdown efficiencies for all preparations were well above 50% (Fig 2C).

Previous studies have investigated the stability of dried-state liposomal preparations at different temperatures (i.e. 37°C to 60°C) over a 1-month period [22], this is the first study to investigate the physiochemical and functional effects of the prolonged storage of lipoplexes over a longer period of time (i.e. 12 months) and at lower temperatures. We developed this lyophilization procedure to ensure effective long-term storage of our lipoplexes, even at RT, as lipoplex suspensions are known to be unstable in aqueous suspension for long-term storage, especially with respect to hydrolysis and size stability. To date no data on the long term (up to 12 months) viability of siRNA-loaded liposomes have, to our knowledge, been published. There are longevity studies investigating traditional liposomes loaded with plasmid DNA. For example, an early study of unextruded liposomes made from DMRIE:DOPE complexed with plasmid DNA showed that when frozen as a aqueous solution they retained activity when thawed 12 months later [23]. More recently it was showed that DC- Cholesterol: DOPE liposomes loaded with plasmid DNA and freeze-dried were stable following 3 months storage at RT and 40°C, although storage at 60°C resulted in lost DNA structure [24]. Others have investigated the stability of DNA oligonucleotide-loaded liposomes for antisense therapies, which changed size and lost ODN content over 12 months (RT) or 90 days (frozen)[25]. However, the functionality of these stored lipoplexes was not examined. Overall, we show that RT was inferior for storage, resulting in an increased particle size and polydispersity from 3 months onwards. Storage at 4°C and -20°C resulted in no changes in lipoplexes and silencing ability. Overall, HFDM liposomes exhibit excellent long-term storage characteristics.

## References

[1] Li SD, Huang L. Targeted delivery of antisense oligodeoxynucleotide and small interference RNA into lung cancer cells. Mol Pharm. 2006;3(5):579–88. 10.1021/mp060039w 17009857

[2] Stuart D, Allen T. A new liposomal formulation for antisense oligodeoxynucleotides with small size, high incorporation efficiency and good stability. Biochimica et Biophysica Acta. 2000;1463:219–29. 10675501 10.1016/s0005-2736(99)00209-6

[3] Wheeler JJ, Palmer L, Ossanlou M, MacLachlan I, Graham RW, Zhang YP, et al Stabilized plasmid-lipid particles: construction and characterization. Gene Ther. 1999;6(2):271–81. 10.1038/sj.gt.3300821 10435112

[4] Maurer N, Wong KF, Stark H, Louie L, McIntosh D, Wong T, et al Spontaneous entrapment of polynucleotides upon electrostatic interaction with ethanol-destabilized cationic liposomes. Biophys J. 2001;80:2310–26. 10.1016/S0006-3495(01)76202-9 11325732 PMC1301421

[5] Jeffs LB, Palmer LR, Ambegia EG, Giesbrecht C, Ewanick S, MacLachlan I. A scalable, extrusion-free method for efficient liposomal encapsulation of plasmid DNA. Pharmaceutical research. 2005;22(3):362–72. 15835741 10.1007/s11095-004-1873-z

[6] Morrissey D, Lockridge J, Shaw L, Blanchard K, Jensen K, Breen W, et al Potent and persistent in vivo anti-HBV activity of chemically modified siRNAs. Nat Biotechnol. 2005;23(8):1002–7. 10.1038/nbt1122 16041363

[7] Li C, Deng Y. A novel method for the preparation of liposomes: Freeze drying of monophase solutions. Journal of Pharmaceutical Sciences. 2004;93(6):1403–14. 10.1002/jps.20055 15124200

[8] Anchordoquy TJ, Carpenter JF, Kroll DJ. Maintenance of transfection rates and physical characterization of lipid/DNA complexes after freeze-drying and rehydration. Arch Biochem Biophys. 1997;348(1):199–206. 10.1006/abbi.1997.0385 9390192

[9] Clement J, Kiefer K, Kimpfler A, Garidel P, Peschka-Suss R. Large-scale production of lipoplexes with long shelf-life. Eur J Pharm Biopharm. 2005;59(1):35–43. 10.1016/j.ejpb.2004.06.001 15567299

[10] Wu SY, Putral LN, Liang M, Chang HI, Davies NM, McMillan NA. Development of a novel method for formulating stable siRNA-loaded lipid particles for in vivo use. Pharmaceutical research. 2009;26(3):512–22. 10.1007/s11095-008-9766-1 19023647

[11] Wu SY, Singhania A, Burgess M, Putral LN, Kirkpatrick C, Davies NM, et al Systemic delivery of E6/7 siRNA using novel lipidic particles and its application with cisplatin in cervical cancer mouse models. Gene Ther. 2011;18(1):14–22. 10.1038/gt.2010.113 20703312

[12] Sheng YH, He Y, Hasnain SZ, Wang R, Tong H, Clarke DT, et al MUC13 protects colorectal cancer cells from death by activating the NF-kappaB pathway and is a potential therapeutic target. Oncogene. 2016. 10.1038/onc.2016.241 PMC5541270 27399336

[13] McCaskill J, Singhania R, Burgess M, Allavena R, Wu S, Blumenthal A, et al Efficient Biodistribution and Gene Silencing in the Lung epithelium via Intravenous Liposomal Delivery of siRNA. Mol Ther Nucleic Acids. 2013;2:e96 10.1038/mtna.2013.22 23736774 PMC3696903

[14] Singhania A, Wu SY, McMillan NA. Effective Delivery of PEGylated siRNA-Containing Lipoplexes to Extraperitoneal Tumours following Intraperitoneal Administration. J Drug Deliv. 2011;2011:192562 10.1155/2011/192562 21773042 PMC3134833

[15] Wu SY, Chang HI, Burgess M, McMillan NA. Vaginal delivery of siRNA using a novel PEGylated lipoplex-entrapped alginate scaffold system. Journal of controlled release: official journal of the Controlled Release Society. 2011;155(3):418–26. 10.1016/j.jconrel.2011.02.002 21315117

[16] Sato A, Takagi M, Shimamoto A, Kawakami S, Hashida M. Small interfering RNA delivery to the liver by intravenous administration of galactosylated cationic liposomes in mice. Biomaterials. 2007;28(7):1434–42. 10.1016/j.biomaterials.2006.11.010 17141864

[17] Merkel OM, Librizzi D, Pfestroff A, Schurrat T, Buyens K, Sanders NN, et al Stability of siRNA polyplexes from poly(ethylenimine) and poly(ethylenimine)-g-poly(ethylene glycol) under in vivo conditions: effects on pharmacokinetics and biodistribution measured by Fluorescence Fluctuation Spectroscopy and Single Photon Emission Computed Tomography (SPECT) imaging. Journal of controlled release: official journal of the Controlled Release Society. 2009;138(2):148–59. 19463870 10.1016/j.jconrel.2009.05.016

[18] Morrissey DV, Lockridge JA, Shaw L, Blanchard K, Jensen K, Breen W, et al Potent and persistent in vivo anti-HBV activity of chemically modified siRNAs. Nat Biotechnol. 2005;23(8):1002–7. 10.1038/nbt1122 16041363

[19] Gabrielli B, Bokhari F, Ranall MV, Oo ZY, Stevenson AJ, Wang W, et al Aurora A Is Critical for Survival in HPV-Transformed Cervical Cancer. Mol Cancer Ther. 2015;14(12):2753–61. 10.1158/1535-7163.MCT-15-0506 26516156

[20] Elbashir SM, Harborth J, Lendeckel W, Yalcin A, Weber K, Tuschl T. Duplexes of 21-nucleotide RNAs mediate RNA interference in cultured mammalian cells. Nature. 2001;411(6836):494–8. 10.1038/35078107 11373684

[21] Bishop CJ, Tzeng SY, Green JJ. Degradable polymer-coated gold nanoparticles for co-delivery of DNA and siRNA. Acta Biomater. 2015;11:393–403. 10.1016/j.actbio.2014.09.020 25246314 PMC4289153

[22] Payton NM, Wempe MF, Xu Y, Anchordoquy TJ. Long-term storage of lyophilized liposomal formulations. Journal of Pharmaceutical Sciences. 2014;103(12):3869–78. 10.1002/jps.24171 25308534 PMC4441342

[23] Zelphati O, Nguyen C, Ferrari M, Felgner J, Tsai Y, Felgner PL. Stable and monodisperse lipoplex formulations for gene delivery. Gene Ther. 1998;5(9):1272–82. 10.1038/sj.gt.3300707 9930330

[24] Yu J, Anchordoquy TJ. Effects of moisture content on the storage stability of dried lipoplex formulations. Journal of Pharmaceutical Sciences. 2009;98(9):3278–89. 10.1002/jps.21846 19569198 PMC2785107

[25] Wyrozumska P, Meissner J, Toporkiewicz M, Szarawarska M, Kuliczkowski K, Ugorski M, et al Liposome-coated lipoplex-based carrier for antisense oligonucleotides. Cancer Biol Ther. 2015;16(1):66–76. 10.4161/15384047.2014.987009 25482931 PMC4329851

